# The Conserved Actinobacterial Two-Component System MtrAB Coordinates Chloramphenicol Production with Sporulation in *Streptomyces venezuelae* NRRL B-65442

**DOI:** 10.3389/fmicb.2017.01145

**Published:** 2017-06-28

**Authors:** Nicolle F. Som, Daniel Heine, Neil A. Holmes, John T. Munnoch, Govind Chandra, Ryan F. Seipke, Paul A. Hoskisson, Barrie Wilkinson, Matthew I. Hutchings

**Affiliations:** ^1^School of Biological Sciences, University of East AngliaNorwich, United Kingdom; ^2^Department of Molecular Microbiology, John Innes CentreNorwich, United Kingdom; ^3^School of Molecular and Cellular Biology, Astbury Centre for Structural Molecular Biology, University of LeedsLeeds, United Kingdom; ^4^Strathclyde Institute of Pharmacy and Biomedical Sciences, University of StrathclydeGlasgow, United Kingdom

**Keywords:** chloramphenicol, cell division, mtrA, *Streptomyces*, antibiotics

## Abstract

*Streptomyces* bacteria make numerous secondary metabolites, including half of all known antibiotics. Production of antibiotics is usually coordinated with the onset of sporulation but the cross regulation of these processes is not fully understood. This is important because most *Streptomyces* antibiotics are produced at low levels or not at all under laboratory conditions and this makes large scale production of these compounds very challenging. Here, we characterize the highly conserved actinobacterial two-component system MtrAB in the model organism *Streptomyces venezuelae* and provide evidence that it coordinates production of the antibiotic chloramphenicol with sporulation. MtrAB are known to coordinate DNA replication and cell division in *Mycobacterium tuberculosis* where TB-MtrA is essential for viability but MtrB is dispensable. We deleted *mtrB* in *S. venezuelae* and this resulted in a global shift in the metabolome, including constitutive, higher-level production of chloramphenicol. We found that chloramphenicol is detectable in the wild-type strain, but only at very low levels and only after it has sporulated. ChIP-seq showed that MtrA binds upstream of DNA replication and cell division genes and genes required for chloramphenicol production. *dnaA*, *dnaN*, *oriC*, and *wblE* (*whiB1*) are DNA binding targets for MtrA in both *M. tuberculosis* and *S. venezuelae*. Intriguingly, over-expression of TB-MtrA and gain of function TB- and Sv-MtrA proteins in *S. venezuelae* also switched on higher-level production of chloramphenicol. Given the conservation of MtrAB, these constructs might be useful tools for manipulating antibiotic production in other filamentous actinomycetes.

## Introduction

*Streptomyces* secondary metabolites account for two thirds of all known antibiotics and numerous other compounds that are used in human medicine as anticancer, antiparasitic, antiviral and immunosuppressant drugs (Devine et al., 2017). Discovery of these natural products peaked in the 1950s but there has been a resurgence of interest in the 21st century, driven by genome sequencing and the increasing threat of drug resistant infections (Katz and Baltz, 2016). Despite their importance however, we still have a poor understanding of how *Streptomyces* bacteria control the production of their secondary metabolites. This is important because ≥ 75% of their secondary metabolite biosynthetic gene clusters (BGCs) are not expressed in laboratory culture and activating cryptic BGCs could facilitate the discovery of new antibiotics and other useful natural products (Hosaka et al., 2009; Antoraz et al., 2015; Rutledge and Challis, 2015).

The major way in which bacteria sense and respond to their environment is through two-component systems and several have been implicated in the regulation of antibiotic production in *Streptomyces* species (Hsiao and Kirby, 2009; Rodriguez et al., 2013). Two component systems typically consist of a bifunctional sensor kinase and a cognate response regulator (Salazar and Laub, 2015). The sensor kinase perceives an extracellular signal and activates its cognate response regulator through a two-step phosphorylation. The phosphorylated regulator (RR∼P) brings about a response to the original signal, usually by modulating target gene expression. In the absence of signal, the bifunctional sensor kinases dephosphorylate their cognate regulators to keep the response switched off (Capra and Laub, 2012). The delicate balance of kinase and phosphatase activities is crucial in modulating the activity of the response regulator and its target genes during the bacterial life cycle (Salazar and Laub, 2015). Cross-talk between two component systems is rare in wild-type cells but removal of a sensor kinase can result in constitutive activation of its response regulator by a non-cognate sensor kinase or the cellular pool of acetyl phosphate (Hutchings, 2007; Capra and Laub, 2012). Similarly, altering a sensor kinase to block its phosphatase activity can result in a response regulator that cannot be dephosphorylated and is rendered constitutively active (Salazar and Laub, 2015).

Here, we report characterisation of the highly conserved actinobacterial two component system MtrAB in the model organism *Streptomyces venezuelae* NRRL B-65442 (Hoskisson and Hutchings, 2006; Supplementary Figure S1). MtrA was first identified as an essential regulator in *M. tuberculosis* (Mycobacterium tuberculosis regulator A) (Zahrt and Deretic, 2000). *M. tuberculosis* MtrA (TB-MtrA) regulates the expression of *dnaA* and *dnaN*, which are essential for DNA replication, and sequesters the origin of DNA replication, *oriC*, in dividing cells. TB-MtrA also regulates the expression of cell division genes and interacts directly with the DnaA protein to prevent the initiation of DNA replication (Purushotham et al., 2015). The TB-MtrB sensor kinase is activated following localisation to the site of cell division through interaction with FtsI and DivIVA (Wag31) and these data have led to a model in which oscillations in TB-MtrA∼P levels play a key role in cell cycle progression with TB-MtrA∼P repressing DNA replication and activating cell division (Plocinska et al., 2012, 2014; Purushotham et al., 2015). An accessory lipoprotein called LpqB modulates the activity of MtrB in mycobacteria and an *M. smegmatis ΔlpqB* mutant grows as *Streptomyces-*like filaments (Nguyen et al., 2010). In the closely related *Corynebacterium glutamicum*, Cg-MtrA is not essential and controls genes involved in cell wall remodeling and the osmotic stress response (Krämer, 2009; Brocker et al., 2011) but deletion of the *mtrAB* genes gives rise to elongated cells which are indicative of a defect in cell division (Möker et al., 2004).

*Streptomyces* bacteria are filamentous saprophytes which grow through the soil as branching substrate mycelium that extends at the hyphal tip (Bush et al., 2015). Nutrient starvation triggers the production of reproductive aerial hyphae that accelerate DNA replication, generating up to 200 copies of the chromosome in each aerial hypha, before undergoing cell division to form chains of unigenomic spores (Bush et al., 2015). Aerial hyphae production and sporulation are coordinated with the production of antibiotics. *S. venezuelae* NRRL B-65442 has recently emerged as a good model for studying development because it completes a full developmental life cycle in liquid growth medium in just 20 h (Munnoch et al., 2016). Here, we present evidence that MtrAB coordinates chloramphenicol production with sporulation in *S. venezuelae*. ChIP-seq shows that MtrA binds directly to genes required for chloramphenicol production and deletion of *mtrB* resulted in a global shift in the metabolome and constitutive, high level production of chloramphenicol. MtrA also shares DNA binding targets with *M. tuberculosis* MtrA (*dnaA*, *dnaN*, *oriC*, and *wblE/whiB1*) and binds to additional cell division genes including *ssgA*, *ssgB*, and *ftsZ*. Expression of TB-MtrA and gain of function TB-MtrA and Sv-MtrA proteins in *S. venezuelae* also activates chloramphenicol production and we propose that manipulating MtrA activity could be a way of upregulating antibiotic production in other *Streptomyces* species.

## Materials and Methods

### Strains, Plasmids, and Primers

The bacterial strains and plasmids used in this study are listed in Supplementary Table S1. Liquid cultures of *Escherichia coli* were routinely grown shaking at 220 rpm, in Lennox Broth (10 g tryptone, 5 g yeast extract, 5 g NaCl made up to 1 L with deionised water) at 37°C. Liquid cultures of *S. venezuelae* NRRL B-65442 were grown in Mannitol Yeast Extract Malt Extract (MYM; Kieser et al., 2000) at 30°C, shaking at 220 rpm with springs. Cultures grown on solid MYM agar were grown at 30°C. To determine the growth rate of *S. venezuelae* strains in liquid culture, a spore inoculum sufficient to reach an OD_600_ of 0.35 after 8 h of growth was added to 35 ml of MYM media in 250 ml flasks containing springs and grown at 30°C at 220 rpm. Absorbance was measured at OD_600_ every 2 h for 20 h and samples were examined with a GXML3000B microscope from GX optical to determine developmental growth phase. Antibacterial bioassays were performed on MYM agar. Mutants were made using the Redirect PCR targeting protocol (Gust et al., 2003) and genes were introduced *in trans* using the integrative vectors pIJ10770 or pIJ10257 (Supplementary Table S1). All strains and plasmids used in this study are either commercially available or available on request. Plasmids were introduced into *S. venezuelae* by conjugation with *Escherichia coli* ET12567/pUZ8002 (Supplementary Table S1) as described (Kieser et al., 2000).

### Immunoblotting

Sv-MtrA-6His was overexpressed in *E. coli* BL21 using the pETDuet vector and purified on an AKTA FPLC system using a 5 mL HisTrap nickel affinity column (GE Healthcare). Polyclonal antiserum was raised against purified Sv-MtrA-6His protein in rabbits by Cambridge Research Biochemicals. Immunoblotting was carried out as described previously (Hutchings et al., 2006b).

### Chromatin ImmunoPrecipitation Followed by Sequencing

Chromatin ImmunoPrecipitation followed by sequencing (ChIP-seq) was performed on *S. venezuelae* NS003 (ΔmtrA ΦBT1 *mtrAp mtrA*-3xFlag; Supplementary Table S1) grown with springs in liquid MYM with wild-type *S. venezuelae* NRRL B-65442 as a control. This strain undergoes a full life cycle in 20 h under these conditions. Samples were taken at 8, 10, 12, 14, 16, 18, and 20 h. ChIP-seq was performed using anti-Flag antibodies (Sigma-Aldrich) and analyzed as described previously (Bush et al., 2013; Crack et al., 2015; Munnoch et al., 2016). Peaks were visually inspected using integrated genome browser (Nicol et al., 2009).

### Method Development and Validation for Chloramphenicol Quantitation

To quantify chloramphenicol, we developed a method based on a high-performance liquid chromatography (HPLC) 1100 system (Agilent Technologies) coupled with a photodiode array detector (DAD). The analysis was performed using a Gemini^®^ 3 μm NX-C18 110 Å at ambient temperature and a flow rate of 0.8 mL/min. We applied a gradient of water/0.1% formic acid/methanol, starting conditions: 90/10, to 0/100 within 14 min, hold for 4 min, to 90/10 within 0.5 min, hold for 1 min. We validated the method as per the International Conference on Harmonization (ICH) guidelines in terms of specificity, sensitivity, linearity, precision, accuracy, and robustness (Supplementary Tables S2, S3).

### Sample Preparation and Analysis

*Streptomyces venezuelae* wild-type, ΔmtrB, and *ermEp^∗^-mtrA* strains were grown in biological triplicates and technical triplicates in MYM medium. An aliquot of 750 μL was taken from each culture and 750 μL of ethyl acetate was added. After shaking for 10 min, the extracts were centrifuged and the organic phase transferred to a 1.5 mL glass vial. The solution was concentrated under reduced pressure and re-dissolved in 500 μL of a mixture of methanol/water (2:3). For quantification 50 μL of each sample was subjected to analytical HPLC. Stock solutions of chloramphenicol for calibration were prepared between a range of 0.01 μg mL^-1^ and 0.05 mg mL^-1^. The stock solutions were extracted and the calibration references prepared in the same way as the actual samples. Generation of the calibration curve and quantification of chloramphenicol was accomplished by integration of the chloramphenicol signal at 273 nm appearing between 10.99 and 11.05 min.

### Method Validation

#### Sensitivity – and Limit of Quantification (LOQ)

The limit of detection (LOD) describes the lowest amount of an analyte in a sample that can be detected but not necessarily quantified. The limit of quantification (LOQ) is the lowest amount of an analyte in a sample that can be determined with suitable precision and accuracy. LOD for the method described here was calculated based on multiplying the noise level by 3 and was found to be 0.008 μg/mL. The LOQ was calculated by multiplying the noise level by 10 and was found to be 0.026 μg/mL. The noise level was determined by measuring a blank sample of methanol. LOD and LOQ were experimentally verified.

#### Linearity and Calibration Curve

Linearity of the method was evaluated by creating a calibration curve as a function of the concentration of the standard analyte chloramphenicol in μg/ml (*X*) and its peak area (*Y*) in the HPLC trace at 273 nm. We found calibration curves to be linear over a concentration range of 0.01–2 μg/mL. The calibration curve gave a good linear regression (*Y* = 164.454*X*-0.0001701, *r*^2^ = 0.9998).

#### Precision and Accuracy

We found the developed method to be precise as the relative standard deviation (RSD) values for repeatability of intra-day and inter-day precision studies were below the limit of 2.5% recommended by the ICH guidelines^10^. To determine the level of accuracy we performed a recovery study by adding three different amounts of a standard to a blank sample. The overall recovery percentages were in the range of 103.2–108.5% which demonstrates a decent accuracy for the quantitation of chloramphenicol.

#### Robustness

Robustness is defined as a measure of the method’s capability to remain unaffected by small, but deliberate variations in some of the methods parameters. We introduced small changes in the HPLC method which may affect the reliability of the method such as flow rate, organic content in mobile phase and wavelength of detection. We observed a low overall RSD between data of varied conditions indicating a good robustness of the method.

### Metabolomics Analysis

We cultivated three individually isolated ΔmtrB mutants and the wild-type in five biological replicates in MYM liquid medium for 3 days. Each sample was analyzed in technical triplicates. An aliquot of 750 μL was taken from each culture and 500 μL of methanol was added. The mixture was shaken for 10 min, centrifuged and 200 μL of the extract transferred to a 1.5 mL glass vial with a 250 μL glass insert. UPLC-HRMS Data was acquired on an Acquity UPLC system (Waters Corporation) equipped with an ACQUITY UPLC^®^ BEH 1.7 μm C18, 1.0 mm × 100 mm column (Waters Corporation) and connected to a Synapt G2-Si high resolution mass spectrometer (Waters Corporation). A gradient of mobile phase A (H_2_O with 0.1% formic acid) and mobile phase B (acetonitrile with 0.1% formic acid) at a flow rate of 80 μL/min was used. Initial conditions were 1% B for 1 min, ramped to 90% B within 6 min, ramped to 100% B within 0.5 min, held for 0.5 min, returned to 1% B within 0.1 min and held for 1.9 min. MS spectra were acquired with a scan time of 1 s in the range of *m*/*z* = 150–1200 in positive MSe-Resolution mode. The following instrument parameters were used: capillary voltage of 3 kV, sampling Cone 40, source offset 80, source temperature 120°C, desolvation temperature 350°C, desolvation gas flow 800 L/h. We used a solution of sodium formate for calibration. A solution of leucine encephalin (H_2_O/MeOH/formic acid: 49.95/49.95/0.1) was used as lock mass and was injected every 15 s during LCMS runs. The lock mass has been acquired with a scan time of 0.3 s and three scans were averaged for each timepoint. The lock mass (*m*/*z* = 556.2766) has been directly applied during data acquisition. All solvents for analytical HPLC and UPLC–MS were obtained commercially at least in HPLC grade from Fisher Scientific and were filtered prior to use. Formic acid (0.1%) was added to the water. We used the Software Progenesis QI (Waters) for processing metabolomics data. The *t*-test (*P* < 0.05) and fold change analysis (fold change > 2) were applied to identify statistically significant differences between the control group (wild-type) and the Δ*mtrB* mutant. EZInfo 3.0 (MKS Umetrics AB) was used for plotting PCA data.

## Results And Discussion

### Deleting *mtrB* Switches on Antibiotic Production in *S. venezuelae* NRRL B65442

**FIGURE 1:**
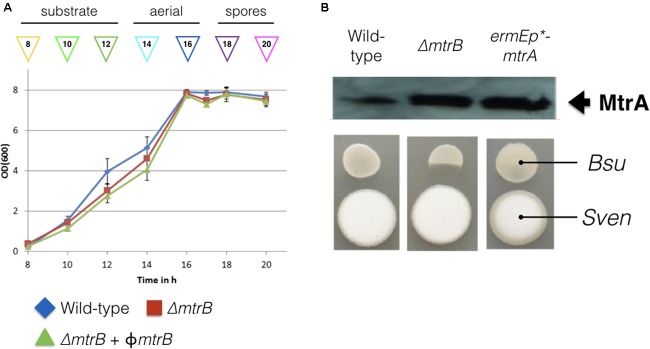
Deletion of *mtrB* has no effect on growth rate but switches on antibacterial activity in *Streptomyces venezuelae* NRRL B-65442. **(A)** Growth curves in liquid MYM medium for the wild-type strain (blue), isogenic ΔmtrB mutant (red) and the ΔmtrB mutant with *mtrB* introduced *in trans* at the ΦBT1 phage integration site under the control of the *mtrAB-lpqB* operon promoter (green). The phases of the life cycle are indicated and the colored arrows show the time points at which samples were taken for ChIP-seq experiments. **(B)** Top panel shows an immunoblot using polyclonal antiserum raised against purified MtrA. Samples were analyzed at 20 h. The lanes contain equal concentrations of total protein from whole cell extracts from the wild-type *S. venezuelae* NRRL B-65442 strain, the isogenic ΔmtrB mutant and the wild-type expressing *mtrA* from the high level, constitutive *ermE^∗^* promoter as verified by Bio-Rad protein assay and Coomassie stained SDS PAGE gels (not shown). Bottom panel. Bioassays of the same strains against *Bacillus subtilis* (Bsu) show that only the *S. venezuelae* (Sven) ΔmtrB mutant has antibacterial activity.

To investigate the function of the MtrAB two component system in *S. venezuelae* we attempted to delete the *mtrA* and *mtrB* genes using a PCR targeting approach (Gust et al., 2003). *mtrB* was readily deleted but we only disrupted the native copy of *mtrA* with a second copy of *mtrA* *in trans*. However, an *mtrA* mutant has been generated in *Streptomyces coelicolor* (Clark et al., 2013) so presumably *mtrA* does not affect viability, but this will require further verification. Deletion of *mtrB* had no effect on growth rate in liquid culture (**Figure 1A**) but immunoblotting with polyclonal MtrA antibodies against wild-type *S. venezuelae* and the ΔmtrB mutant suggests that MtrA is over-produced in the absence of MtrB and to the same levels as when *mtrA* is expressed from the high level, constitutive *ermE^∗^* promoter (**Figure 1B**). Phenotypic screening of the ΔmtrB mutant also revealed that it inhibits the growth of *Bacillus subtilis* whereas neither the wild-type strain or the wild-type expressing *ermEp^∗^*-*mtrA* has antibacterial activity (**Figure 1B**). The simplest explanation of these data is that deletion of *mtrB* results in constitutive phosphorylation of MtrA and that MtrA∼P then constitutively activates expression of its target genes. We previously reported a similar result for the *S. coelicolor* response regulator VanR which is constitutively active in the absence of its cognate sensor kinase, VanS (Hutchings et al., 2006a). VanR and MtrA both belong to the OmpR family of response regulators and activation of the DNA binding activity in OmpR family proteins by phosphorylation is well-established (Friedland et al., 2007).

### The *S. venezuelae* ΔmtrB Strain Constitutively Produces Chloramphenicol

**FIGURE 2:**
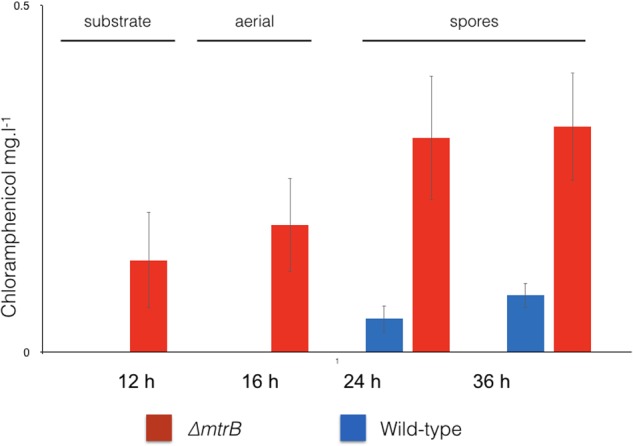
Chloramphenicol is produced constitutively and at higher levels in the absence of MtrB. Levels of chloramphenicol detected in wild-type *S. venezuelae* NRRL B-65442 (blue) and the isogenic ΔmtrB mutant (red). Note that chloramphenicol is only produced following sporulation in the wild-type but is produced at all stages of growth and at higher levels in the ΔmtrB mutant. All measurements were on biological and technical triplicate samples.

We reasoned that the antibacterial activity switched on in the *mtrB* mutant is most likely due to the production of chloramphenicol, which is reported to be switched off in wild-type *S. venezuelae* NRRL B-65442 (Fernández-Martínez et al., 2014). To further investigate this, we measured production of chloramphenicol in biological and technical triplicates of the wild-type strain and the ΔmtrB mutant after 12, 16, 24, and 36 h of growth in liquid MYM medium (**Figure 2**). Consistent with the link between antibiotic production and sporulation in streptomycetes, we could detect low levels of chloramphenicol in cultures of the wild-type strain but only after it had sporulated (**Figure 2**). Cultivation for 24 h in biological and technical triplicates confirmed an increased production of chloramphenicol in the ΔmtrB mutant with a mean concentration of 0.41 mg/L which is >30 times higher than the wild-type strain (0.013 mg/L) or wild-type over-expressing *mtrA* (0.010 mg/L). Importantly, chloramphenicol production is constitutive in the *S. venezuelae ΔmtrB* strain. Chloramphenicol biosynthesis is presumably switched on much earlier in the growth cycle of the ΔmtrB strain because we detect the antibiotic in actively growing cultures (12 h) and cultures that are differentiating into aerial hyphae and spores (16–36 h, **Figures 1**, **2**).

### Deletion of *mtrB* Results in a Global Shift in the Metabolome

**FIGURE 3:**
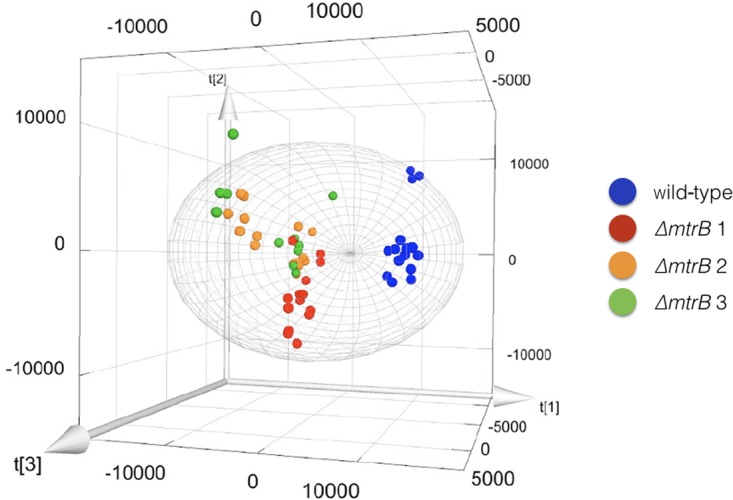
Loss of MtrB results in a global shift in the *S. venezuelae* NRRL B-65442 metabolome. Principal component analysis on the wild-type (blue dots) and three independently isolated *mtrB* mutants (red, green, and orange dots). Data from *ΔmtrB* mutant strains clearly group together, and are distinct from data obtained from the wild-type while variations within each group are comparably small.

To examine the effects of deleting *mtrB* on the metabolome of *S. venezuelae*, we cultivated the wild-type strain and three independently isolated *ΔmtrB* mutants in five biological replicates each in technical triplicates and analyzed the extracts by UPLC/HRMS using untargeted metabolomics. Runs were aligned to compensate for between-run variation and a peak-picking algorithm was applied to allow for the immaculate matching of each feature (a discrete *m*/*z* value and its retention time) among all runs. Following normalization, features could be compared quantitatively and their putative identity proposed based on their high-resolution MS-signal. Comparing the level of metabolite signals, it appeared that all three *ΔmtrB* mutants showed an extensive alteration of global metabolite levels. To display multidimensional data, we used principal component analysis (**Figure 3**). Each sphere in the 3D Plot represents one dataset obtained from a single UPLC-HRMS run. Data from the individual *ΔmtrB* mutant strains clearly group together, and are distinct from data obtained from the wild-type, while variations within each group are comparably small. The 3D Plot therefore shows consistent and global changes in the metabolome upon loss of MtrB (**Figure 3**).

### MtrA Binds Directly to Genes Required for Chloramphenicol Production

**FIGURE 4:**
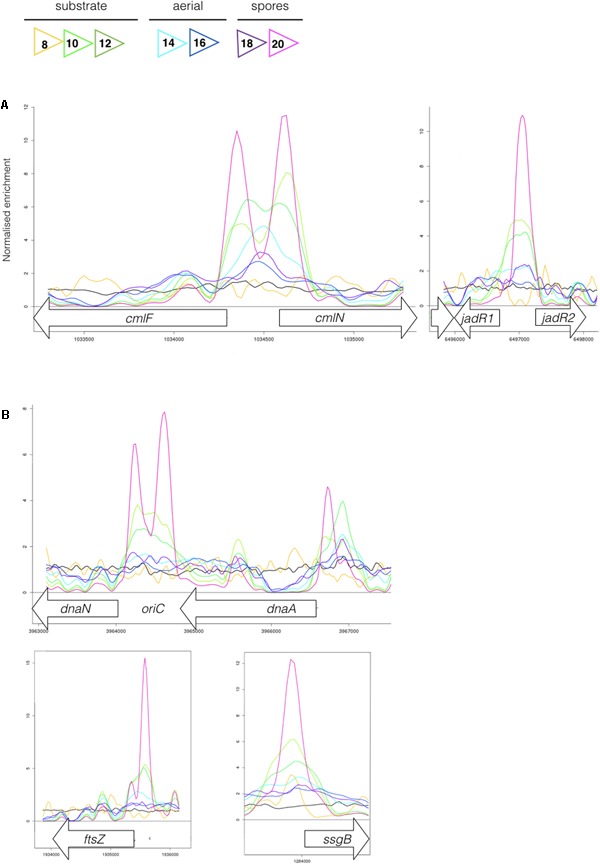
**(A)** MtrA binds upstream of genes affecting chloramphenicol production. The colored lines show ChIP-seq data for MtrA-3xFlag at 8 h (yellow), 10 h (light green), 12 h (dark green), 14 h (light blue), 16 h (dark blue), 18 h (purple), and 20 h (pink), see **Figure 1** for life cycle stages. The black line is the wild-type (negative) control. ChIP peaks are shown at the divergent *cmlF* and *cmlN* genes, which encode transporters required for chloramphenicol production (Fernández-Martínez et al., 2014), and *jadR1* and *jadR2* genes, which encode transcription factors that cross-regulate the jadomycin and chloramphenicol biosynthetic gene clusters (BGCs) (Liu et al., 2013). **(B)** MtrA binds upstream of genes required for DNA replication and cell division. ChIP-seq peaks, as in **Figure 4**, upstream of the DNA replication genes *dnaN* and *dnaA* and the cell division genes *ftsZ* and *ssgB.* In actinomycetes the origin of DNA replication, *oriC*, is between the *dnaN* and *dnaA* genes.

**FIGURE 5:**
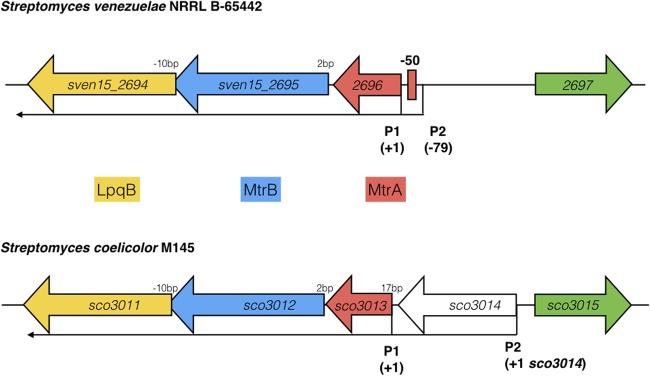
Arrangement of the *mtrAB-lpqB* operon in *S. venezuelae* NRRL B-65442 and *Streptomyces coelicolor* M145. Published dRNA-seq data (Jeong et al., 2016; Munnoch et al., 2016) shows that the major transcriptional start site is at +1 in both species, relative to the *mtrA* translational start codon. A second transcript starts at –79 in *S. venezuelae* and an MtrA ChIP peak is centered between P1 and P2 at –50, suggesting autoregulation. In *S. coelicolor* a gene encoding a putative eukaryotic translation initiation factor has inserted immediately upstream of *mtrA* and is also expressed on a leaderless transcript.

Most sensor kinases are bifunctional and act as phosphatases in the absence of signal. This prevents aberrant activation of their response regulators through cross-talk. Removal of a sensor kinase can lead to activation of a response regulator by either the cellular pool of acetyl phosphate or by non-cognate sensor kinases. We hypothesize that loss of MtrB results in constitutive phosphorylation of MtrA and activation of its target genes and it seemed likely that these targets include genes in the chloramphenicol BGC. To test this, we performed ChIP-seq against a strain expressing a C-terminally 3xFlag-tagged MtrA protein from its native promoter, integrated at the ΦBT1 site, with the native *mtrA* gene deleted. The fact we could delete *mtrA* in a strain carrying this construct suggested that MtrA-3xFlag is functional and the strain grew like the wild-type (Supplementary Figure S2). We performed ChIP-seq on cultures grown for 8, 10, 12, 14, 16, 18, and 20 h in liquid MYM medium (NCBI Geo database accession number GSE84311; **Figure 1**; Som et al., 2016). Reads were mapped to the *S. venezuelae* NRRL B-65442 genome (GenBank accession number CP018074) and the chloramphenicol BGC was examined for MtrA ChIP peaks. The data show significant enrichment (cut-off *P* < 0.05) between the divergent *cmlN* and *cmlF* genes in the chloramphenicol BGC, which encode transporters required for chloramphenicol production (Fernández-Martínez et al., 2014). This indicates direct binding by MtrA (**Figure 4A**). MtrA also binds between the divergent *jadR1* and *jadR2* genes in the jadomycin BGC and this may affect chloramphenicol production. JadR1 activates and JadR2 represses jadomycin biosynthesis but they also cross regulate the chloramphenicol BGC in other *S. venezuelae* strains (Zhang et al., 2013; Forget et al., 2017). JadR1 represses expression of the chloramphenicol BGC and is itself repressed by JadR2 (Liu et al., 2013) so MtrA could feasibly activate JadR2 and/or repress JadR1 since it is known to act as both activator and repressor in other actinomycetes. However, we do not yet know if MtrA regulates expression of these genes. Under the conditions used we did not detect any jadomycins in the wild-type or ΔmtrB strains using LCMS with verified jadomycin standards. However, the phenotype of the ΔmtrB mutant suggests that MtrB is required to coordinate chloramphenicol production with sporulation in *S. venezuelae*, presumably through phosphorylation/dephosphorylation of MtrA. Consistent with this we found that, as in *M. tuberculosis*, *S. venezuelae* MtrA binds to essential targets involved in DNA replication and cell division (**Figure 4B** and **Table 1**). The MtrA targets shared between *M. tuberculosis* and *S. venezuelae* are *oriC*, *dnaA*, *dnaN*, and *wblE*/*whiB1* (**Table 1**) (Purushotham et al., 2015). *oriC* is the origin of DNA replication in bacteria and is bound by multiple DnaA molecules which melt the DNA to initiate DNA replication. In actinomycetes *oriC* falls in the intergenic region between *dnaN* and *dnaA*. *M. tuberculosis* MtrA sequesters *oriC* by binding to three sites and also represses expression of *dnaA* and *dnaN* (Purushotham et al., 2015). The *wblE* gene encodes a member of WhiB-like (Wbl) family of transcription factors, which is restricted to actinomycetes. They are not well-understood but they appear to all contain 4Fe–4S iron sulfur clusters and nitrosylation of this cluster in the *M. tuberculosis* WblE homolog, WhiB1, activates the protein for DNA binding (Smith et al., 2012). The complete *M. tuberculosis* MtrA regulon has not been published so more targets may be conserved. MtrA likely autoregulates its own expression because there is an MtrA ChIP peak centered at -50 relative to the major transcription start site at +1, as determined by examining published differential RNA sequencing data (dRNA-seq; Supplementary Figure S3) (Munnoch et al., 2016). The position of this MtrA binding site is consistent with auto-activation and could explain why the MtrA protein appears to be over-produced in the ΔmtrB strain (**Figure 1**). A second, minor transcript (P2) initiates upstream of the MtrA binding site at -79 bp (**Figure 5** and Supplementary Figure S2). Published dRNA-seq data for *S. coelicolor* also shows that the *mtrAB-lpqB* gene are expressed on a leaderless transcript starting at +1 relative to the *mtrA* translation start codon (Jeong et al., 2016). However, in *S. coelicolor* another gene, encoding the putative eukaryotic translation initiation factor Sco3014, has inserted immediately upstream of *mtrA* and P2 starts at +1 relative to the *sco3014* translation start codon (**Figure 5**).

**Table 1:** Chromatin ImmunoPrecipitation followed by sequencing (ChIP-seq) data against MtrA-3xFlag.

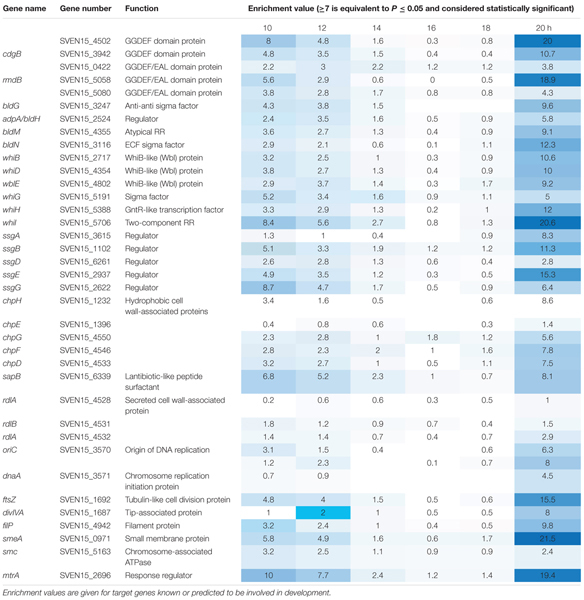

### *M. tuberculosis* MtrA Switches on Chloramphenicol Production in *S. venezuelae*

**FIGURE 6:**
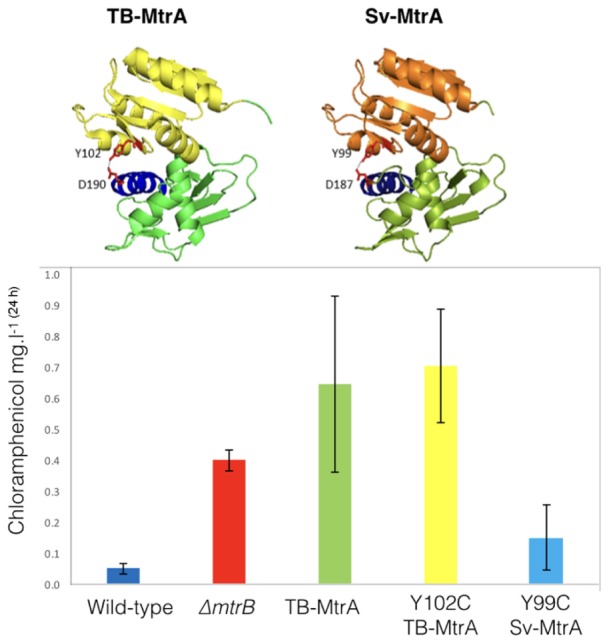
Activating chloramphenicol production using TB- and Sv-MtrA expression constructs. **(Top)** Modeling *S. venezuelae* MtrA (Sv-MtrA) on the published *Mycobacterium tuberculosis* MtrA (TB-MtrA) structure (Friedland et al., 2007) suggests they are likely to be highly similar. **(Bottom)** Expression of TB-MtrA and the gain of function Y102C TB-MtrA proteins in wild-type *S. venezuelae* NRRL B-65442 switches on high level production of chloramphenicol. The equivalent Y99C change in Sv-MtrA has a smaller effect on chloramphenicol production.

Sv-MtrA shares 75% primary sequence identity and several target genes with TB-MtrA so we were curious to see if expression of TB-MtrA or the gain-of-function Y102C TB-MtrA in *S. venezuelae* would switch on chloramphenicol production. We also modeled Sv-MtrA onto the published TB-MtrA structure using HHrep (Soding et al., 2006; Friedland et al., 2007) and made the equivalent Y99C (predicted gain-of-function) change in Sv-MtrA (**Figure 6**). We made expression constructs for all three, driven by the *S. venezuelae mtrA* promoter, and introduced them into the ΦBT1 phage integration site (Gregory et al., 2003). All three strains grew normally so we measured chloramphenicol production in these strains and found that they all produced significantly more chloramphenicol than the wild-type (**Figure 6**) or wild-type over-expressing *mtrA* from the *ermE^∗^* promoter (see above). It is puzzling that wild-type TB-MtrA has such a large effect and our only explanation is that TB-MtrA may be constitutively phosphorylated in *S. venezuelae* and not subject to control by Sv-MtrB.

## Conclusion

In this study, we have carried out the first characterisation of MtrAB in the genus *Streptomyces*, using the model organism *S. venezuelae* NRRL B65542. ChIP-seq at regular time points throughout the life cycle indicate that the DNA binding activity of Sv-MtrA is developmentally regulated and this, combined with the fact that Sv-MtrA binds to many developmental genes (**Table 1**) suggests MtrAB plays a role in regulating life cycle progression in *S. venezuelae*. MtrA activity changes during the life cycle and appears to be biphasic, being most active in vegetatively growing substrate hyphae and in spores but less active in aerial hyphae that are undergoing rapid DNA replication. This is most likely because MtrA is subject to developmental regulation via phosphorylation/dephosphorylation by MtrB so it will be important to discover how MtrB activity is regulated, e.g., by interaction with the cell division machinery as in *M. tuberculosis* (Plocinska et al., 2012, 2014). One key role uncovered here for MtrAB is the coordination of chloramphenicol production with sporulation. We have shown that deletion of *mtrB* uncouples these processes and results in constitutive production of this antibiotic. It is perhaps surprising that we do not see any effects on sporulation in the ΔmtrB strain but the developmental genes bound by Sv-MtrA are subject to complex regulation which presumably cannot be over-ridden simply by activating MtrA (Bush et al., 2015). Nevertheless, we have shown that manipulating the activity of MtrAB proteins can be used to upregulate antibiotic production and given their conservation in filamentous actinomycetes this may provide another useful tool to switch on cryptic BGCs. Future work will focus on testing whether MtrA is essential, if and how it regulates the gene targets identified using ChIP-seq and understanding how MtrB activity is regulated in *Streptomyces* species.

## Author Contributions

NS carried out all the molecular microbiology experiments, DH carried out all the natural product chemistry experiments, NH and RS made strains and constructs for this study, JM and GC analyzed ChIP- and RNA-seq data. PH, BW, and MH conceived the project and analyzed data and all authors wrote the manuscript.

## Conflict of Interest Statement

The authors declare that the research was conducted in the absence of any commercial or financial relationships that could be construed as a potential conflict of interest.

## References

[B1] Antoraz S. Santamaria R. I. Diaz M. Sanz D. Rodriguez H. (2015). Toward a new focus in antibiotic and drug discovery from the *Streptomyces arsenal*. *Front. Microbiol.* 6:461 10.3389/fmicb.2015.00461 PMC4429630 26029195

[B2] Brocker M. Mack C. Bott M. (2011). Target genes, consensus binding site, and role of phosphorylation for the response regulator MtrA of *Corynebacterium glutamicum*. *J. Bacteriol.* 193 1237–1249. 10.1128/JB.01032-10 21183673 PMC3067596

[B3] Bush M. J. Bibb M. J. Chandra G. Findlay K. C. Buttner M. J. (2013). Genes required for aerial growth, cell division, and chromosome segregation are targets of WhiA before sporulation in *Streptomyces venezuelae*. *mBio* 4:e00684-13. 10.1128/mBio.00684-13 PMC3781837 24065632

[B4] Bush M. J. Tschowri N. Schlimpert S. Flärdh K. Buttner M. J. (2015). c-di-GMP signalling and the regulation of developmental transitions in streptomycetes. *Nat. Rev. Microbiol.* 13 749–760. 10.1038/nrmicro3546 26499894

[B5] Capra E. J. Laub M. T. (2012). Evolution of two-component signal transduction systems. *Annu. Rev. Microbiol.* 66 325–347. 10.1146/annurev-micro-092611-150039 22746333 PMC4097194

[B6] Clark L. C. Seipke R. F. Prieto P. Willemse J. van Wezel G. P. Hutchings M. I. (2013). Mammalian cell entry genes in *Streptomyces* may provide clues to the evolution of bacterial virulence. *Sci. Rep.* 3:1109 10.1038/srep01109 PMC3552289 23346366

[B7] Crack J. C. Munnoch J. Dodd E. L. Knowles F. Al Bassam M. M. Kamali S. (2015). NsrR from *Streptomyces coelicolor* is a nitric oxide-sensing [4Fe-4S] cluster protein with a specialized regulatory function. *J. Biol. Chem.* 290 12689–12704. 10.1074/jbc.M115.643072 25771538 PMC4432287

[B8] Devine R. Hutchings M. I. Holmes N. A. (2017). Future directions for the discovery of antibiotics from actinomycete bacteria. *Emerg. Top. Life Sci.* 1 1–12. 10.1042/ETLS20160014 33525817

[B9] Fernández-Martínez L. T. Borsetto C. Gomez-Escribano J. P. Bibb M. J. Al-Bassam M. M. Chandra G. (2014). New insights into chloramphenicol biosynthesis in *Streptomyces venezuelae* ATCC 10712. *Antimicrob. Agents Chemother.* 58 7441–7450. 10.1128/AAC.04272-14 25267678 PMC4249514

[B10] Forget S. M. McVey J. Vining L. C. Jakeman D. L. (2017). *Streptomyces venezuelae* ISP5230 maintains excretion of jadomycin upon disruption of the MFS transporter JadL located within the natural product biosynthetic gene cluster. *Front. Microbiol.* 8:432 10.3389/fmicb.2017.00432 PMC5359229 28377749

[B11] Friedland N. Mack T. R. Yu M. Hung L.-W. Terwilliger T. C. Waldo G. S. (2007). Domain orientation in the inactive response regulator *Mycobacterium tuberculosis* MtrA provides a barrier to activation. *Biochemistry* 46 6733–6743. 10.1021/bi602546q 17511470 PMC2528954

[B12] Gregory M. A. Till R. Smith M. C. M. (2003). Integration site for *Streptomyces* phage phiBT1 and development of site-specific integrating vectors. *J. Bacteriol.* 185 5320–5323. 10.1128/JB.185.17.5320-5323.2003 12923110 PMC180994

[B13] Gust B. Challis G. L. Fowler K. Kieser T. Chater K. F. (2003). PCR-targeted *Streptomyces* gene replacement identifies a protein domain needed for biosynthesis of the sesquiterpene soil odor geosmin. *Proc. Natl. Acad. Sci. U.S.A.* 100 1541–1546. 10.1073/pnas.0337542100 12563033 PMC149868

[B14] Hosaka T. Ohnishi-Kameyama M. Muramatsu H. Murakami K. Tsurumi Y. Kodani S. (2009). Antibacterial discovery in actinomycetes strains with mutations in RNA polymerase or ribosomal protein S12. *Nat. Biotechnol.* 27 462–464. 10.1038/nbt.1538 19396160

[B15] Hoskisson P. A. Hutchings M. I. (2006). MtrAB-LpqB: a conserved three-component system in actinobacteria? *Trends Microbiol.* 14 444–449. 10.1016/j.tim.2006.08.005 16934981

[B16] Hsiao N.-H. Kirby R. (2009). Two-component signal transduction systems in *Streptomyces* and related organisms studied using DNA comparative microarray analysis. *Antonie Van Leeuwenhoek* 95 189–206. 10.1007/s10482-008-9302-7 19151927

[B17] Hutchings M. I. (2007). Unusual two-component signal transduction pathways in the actinobacteria. *Adv. Appl. Microbiol.* 61 1–26. 10.1016/S0065-2164(06)61001-0 17448786

[B18] Hutchings M. I. Hong H.-J. Buttner M. J. (2006a). The vancomycin resistance VanRS two-component signal transduction system of *Streptomyces coelicolor*. *Mol. Microbiol.* 59 923–935. 10.1111/j.1365-2958.2005.04953.x 16420361

[B19] Hutchings M. I. Hong H.-J. Leibovitz E. Sutcliffe I. C. Buttner M. J. (2006b). The sigma(E) cell envelope stress response of *Streptomyces coelicolor* is influenced by a novel lipoprotein. CseA. *J. Bacteriol.* 188 7222–7229. 10.1128/JB.00818-06 17015661 PMC1636229

[B20] Jeong Y. Kim J.-N. Kim M. W. Bucca G. Cho S. Yoon Y. J. (2016). The dynamic transcriptional and translational landscape of the model antibiotic producer *Streptomyces coelicolor* A3(2). *Nat. Commun.* 7:11605 10.1038/ncomms11605 PMC4895711 27251447

[B21] Katz L. Baltz R. H. (2016). Natural product discovery: past, present, and future. *J. Ind. Microbiol. Biotechnol.* 43 155–176. 10.1007/s10295-015-1723-5 26739136

[B22] Kieser T. Bibb M. J. Buttner M. J. Chater K. F. Hopwood D. A. (2000). *Practical Streptomyces Genetics.* Bungay: John Innes Foundation.

[B23] Krämer R. (2009). Osmosensing and osmosignaling in *Corynebacterium glutamicum*. *Amino Acids* 37 487–497. 10.1007/s00726-009-0271-6 19308662

[B24] Liu G. Chater K. F. Chandra G. Niu G. Tan H. (2013). Molecular regulation of antibiotic biosynthesis in *Streptomyces*. *Microbiol. Mol. Biol. Rev.* 77 112–143. 10.1128/MMBR.00054-12 23471619 PMC3591988

[B25] Möker N. Brocker M. Schaffer S. Krämer R. Morbach S. Bott M. (2004). Deletion of the genes encoding the MtrA-MtrB two-component system of *Corynebacterium glutamicum* has a strong influence on cell morphology, antibiotics susceptibility and expression of genes involved in osmoprotection. *Mol. Microbiol.* 54 420–438. 10.1111/j.1365-2958.2004.04249.x 15469514

[B26] Munnoch J. T. Martinez M. T. P. Svistunenko D. A. Crack J. C. Le Brun N. E. Hutchings M. I. (2016). Characterization of a putative NsrR homologue in *Streptomyces venezuelae* reveals a new member of the Rrf2 superfamily. *Sci. Rep.* 6:31597 10.1038/srep31597 PMC5015018 27605472

[B27] Nguyen H. T. Wolff K. A. Cartabuke R. H. Ogwang S. Nguyen L. (2010). A lipoprotein modulates activity of the MtrAB two-component system to provide intrinsic multidrug resistance, cytokinetic control and cell wall homeostasis in *Mycobacterium*. *Mol. Microbiol.* 76 348–364. 10.1111/j.1365-2958.2010.07110.x 20233304

[B28] Nicol J. W. Helt G. A. Blanchard S. G. Raja A. Loraine A. E. (2009). The integrated genome browser: free software for distribution and exploration of genome-scale datasets. *Bioinformatics* 25 2730–2731. 10.1093/bioinformatics/btp472 19654113 PMC2759552

[B29] Plocinska R. Martínez L. Gorla P. Pandeeti E. Sarva K. Blaszczyk E. (2014). *Mycobacterium tuberculosis* MtrB sensor kinase interactions with FtsI and Wag31 proteins reveal a role for MtrB distinct from that regulating MtrA activities. *J. Bacteriol.* 196 4120–4129. 10.1128/JB.01795-14 25225272 PMC4248866

[B30] Plocinska R. Purushotham G. Sarva K. Vadrevu I. S. Pandeeti E. V. P. Arora N. (2012). Septal localization of the *Mycobacterium tuberculosis* MtrB sensor kinase promotes MtrA regulon expression. *J. Biol. Chem.* 287 23887–23899. 10.1074/jbc.M112.346544 22610443 PMC3390665

[B31] Purushotham G. Sarva K. B. Blaszczyk E. Rajagopalan M. Madiraju M. V. (2015). *Mycobacterium tuberculosis* oriC sequestration by MtrA response regulator. *Mol. Microbiol.* 98 586–604. 10.1111/mmi.13144 26207528 PMC4700885

[B32] Rodriguez H. Rico S. Diaz M. Santamaria R. I. (2013). Two-component systems in *Streptomyces*: key regulators of antibiotic complex pathways. *Microb. Cell Fact.* 12:127 10.1186/1475-2859-12-127 PMC3881020 24354561

[B33] Rutledge P. J. Challis G. L. (2015). Discovery of microbial natural products by activation of silent biosynthetic gene clusters. *Nat. Rev. Microbiol.* 13 509–523. 10.1038/nrmicro3496 26119570

[B34] Salazar M. E. Laub M. T. (2015). Temporal and evolutionary dynamics of two-component signaling pathways. *Curr. Opin. Microbiol.* 24 7–14. 10.1016/j.mib.2014.12.003 25589045 PMC4380680

[B39] Smith L. J. Stapleton M. R. Buxton R. S. Green J. (2012). Structure-function relationships of the *Mycobacterium tuberculosis* transcription factor WhiB1. *PLoS ONE* 7:e40407. 10.1371/journal.pone.0040407 PMC3390391 22792304

[B35] Soding J. Remmert M. Biegert A. (2006). HHrep: de novo protein repeat detection and the origin of TIM barrels. *Nucleic Acids Res.* 34 W137–W142. 10.1093/nar/gkl130 16844977 PMC1538828

[B36] Som N. F. Heine D. Munnoch J. T. Holmes N. A. Knowles F. Chandra G. (2016). MtrA is an essential regulator that coordinates antibiotic production and development in *Streptomyces* species. *bioRxiv* 10.1101/090399

[B37] Zahrt T. C. Deretic V. (2000). An essential two-component signal transduction system in *Mycobacterium tuberculosis*. *J. Bacteriol.* 182 3832–3838. 10.1128/JB.182.13.3832-3838.2000 10851001 PMC94557

[B38] Zhang Y. Pan G. Zou Z. Fan K. Yang K. Tan H. (2013). JadR^∗^-mediated feed-forward regulation of cofactor supply in jadomycin biosynthesis. *Mol. Microbiol.* 90 884–897. 10.1111/mmi.12406 24112541

