# Supplementary material for: The Conserved Actinobacterial Two-Component System MtrAB Coordinates Chloramphenicol Production with Sporulation in Streptomyces venezuelae NRRL B-65442
Source: Front Microbiol. 2017 Jun 28;8:1145. doi: 10.3389/fmicb.2017.01145 (PMC5487470; doi:10.3389/fmicb.2017.01145)
Supplement: Supplementary file 1 [file Table_1.docx]

**The conserved actinobacterial two-component system MtrAB coordinates chloramphenicol production with sporulation in *Streptomyces venezuelae* NRRL B-65442**

Nicolle F. Som^1#^, Daniel Heine^2#^, Neil A. Holmes^1^, John T. Munnoch^1^, Govind Chandra^2^, Ryan F. Seipke^1,3^, Paul A. Hoskisson^4^, Barrie Wilkinson^2*^ and Matthew I Hutchings^1*^

# Authors contributed equally; * Correspondence

^1^School of Biological Sciences, University of East Anglia, Norwich Research Park, Norwich, United Kingdom. NR4 7TJ.

^2^Department of Molecular Microbiology, John Innes Centre, Norwich Research Park, Norwich, United Kingdom. NR4 7TJ.

^3^ School of Molecular & Cellular Biology, Astbury Centre for Structural Molecular Biology, University of Leeds, Leeds, LS2 9JT, UK

^4^Strathclyde Institute of Pharmacy and Biomedical Sciences, University of Strathclyde, 161, Cathedral Street, Glasgow, G4 0RE, UK

**Table S1.** Strains and plasmids used in this work. All plasmids made in this work are available on request from [m.hutchings@uea.ac.uk](mailto:m.hutchings@uea.ac.uk).

| **Strains used in this work** | | |
| --- | --- | --- |
| **Bacterial strain** | **Description** | **Reference** |
| *E. coli* Top10 | F– mcrA Δ(mrr-hsdRMS-mcrBC) Φ80lacZΔM15 ΔlacX74 recA1 araD139 Δ(ara leu) 7697 galU galK rpsL (StrR) endA1 nupG | Invitrogen |
| *E. coli* BW25113 | λ^-^, Δ(*araD-araB*)*567,* Δ*lacZ4787*(*::rrnB-4*)*, lacIp-4000*(*lacIQ*), *rpoS369*(*Am*)*, rph-1,* Δ(*rhaD-rhaB*)*568, hsdR514* | ^4^ |
| *E. coli* ET12567 | *dam^-^ dcm^-^ hsdS^-^* | ^4^ |
| *E. coli* BL21 | *fhuA2 [lon] ompT gal (λ DE3) [dcm] ∆hsdSλ DE3 = λ sBamHIo ∆EcoRI-B int::(lacI::PlacUV5::T7 gene1) i21 ∆nin5* | New England Biolabs |
| *S. venezuelae* NRRL B-65442 | *S. venezuelae* wild-type strain | USDA ARS Culture Collection. <https://nrrl.ncaur.usda.gov/cgi-bin/usda> Strain no. [B-65442](https://nrrl.ncaur.usda.gov/cgi-bin/usda/prokaryote/report.html?nrrlcodes=B%2d65442). |
| NS003 | *S. venezuelae* NRRL B65442*∆mtrA* ΦBT1 *mtrAp mtrA*-3xFlag | This work |
| NS012, NS021, NS022 | *S. venezuelae* NRRL B65442 *∆mtrB*::*aac(3)IV oriT* | This work |
| NS013 | *S. venezuelae* NRRL B65442 ΦBT1 *mtrAp*-*mtrA*(TB) | This work |
| NS016 | *S. venezuelae* NRRL B65442 ΦBT1*mtrAp*-*mtrA*(TB) _Y102C_ | This work |
| NS029 | *S. venezuelae* NRRL B65442 ΦBT1 pMS82 | This work |
| NS033 | *S. venezuelae* NRRL B65442 ΦBT1 *ermE* mtrA* | This work |
| NS036 | *S. venezuelae* NRRL B65442 ΦBT1 pIJ10257 | This work |
| NS039 | *S. venezuelae* NRRL B65442 ΦBT1 *mtrAp-mtrA* | This work |
| NS052 | *S. venezuelae* NRRL B65442 ∆*mtrA*:: *aac(3)IV oriT* ΦBT1 *mtrAp-mtrA* | This work |
| NS093 | *S. venezuelae* NRRL B65442 ∆*mtrB*:: *aac(3)IV oriT* ΦBT1 *mtrAp-mtrB* | This work |
| NS099 | *S. venezuelae* NRRL B65442 ΦBT1 *mtrAp*-*mtrA*_Y99C_ | This work |
| SP001 | *S. venezuelae* NRRL B65442 ΦBT1 pSP001 | This work |
| SP002 | *S. venezuelae* NRRL B65442 ΦBT1 pSP002 | This work |
| SP003 | *S. venezuelae* NRRL B65442 ΦBT1 pSP003 | This work |
| **Cosmids used in this work** | | |
| **Cosmid** | **Description** | **Reference** |
| SV-6-A04 | Supercos-1 Cosmid with a 40.2kbp chromosomal fragment with *mtrAB-lpqB* | John Innes Centre, Norwich, UK. |
| pNS070 | SV-6-A04 *mtrB*::*aac(3)IV oriT* | This work |
| pNS071 | SV-6-A04 *mtrA*::*aac(3)IV oriT* | This work |
| **Plasmids used in this work** | | |
| **Plasmid** | **Description** | **Reference** |
| pIJ773 | *aac(3)IV oriT bla* | ^4^ |
| pIJ790 | *araC-Parab,Υ, β, exo, cat, repA1001ts, oriR101* | ^4^ |
| pUZ8002 | RK2 derivative with a mutation in *oriT* | ^4^ |
| pMS82 | *ori,* pUC18, *hyg, oriT,* RK2, int ΦBT1 | ^5^ |
| pETDuet |  | Novagen |
| pNS074 | pETDuet + Sv-*mtrA* | This work |
| pIJ10770 | *ori,* pUC18, *hyg, oriT,* RK2, int ΦBT1 Δ *aac(3)IVp* (pMS82 derivative with the *aac* promoter deleted). | Schlimpert and Buttner, in prep. |
| pIJ10257 | *oriT*, ΦBT1 *attB-int*, Hygr, *ermEp**, pMS81 backbone | ^2^ |
| pNS102 | pIJ10257 *mtrA* | This work |
| pNS103 | pIJ10770 *mtrAp mtrA*(TB) | This work |
| pNS104 | pIJ10770 *mtrAp mtrA*(TB)_Y102C_ | This work |
| pNS105 | pIJ10770 *mtrAp mtrA*(Sv)_Y99C_ | This work |
| pNS106 | pMS82 *mtrAp lpqB* | This work |
| pNS107 | pIJ10770 *mtrAp mtrB* | This work |
| pNS108 | pMS82 *mtrAp mtrA* | This work |
| pNS109 | pMS82 *mtrAp mtrA*-3xFlag | This work |

**Table S2**. Intra-day and inter-day precision data of the HPLC method (*n* = 3).

| Standard (µg/mL) | Intra-day (RSD %) | Inter-day (RSD %) |
| --- | --- | --- |
| 0.1 | 1.48 | 1.64 |
| 0.5 | 0.59 | 1.16 |
| 1 | 0.24 | 0.16 |

**Table S3**. Analytical recovery of Chloramphenicol by developed HPLC method (*n* = 3).

| Added concentration (µg/mL) | Measured concentration  (µg/mL) | Recovery (%) | RSD (%) | Mean recovery (%) |
| --- | --- | --- | --- | --- |
| 0.1 | 0.109 | 108.5 | 0.48 | 105.0 |
| 0.5 | 0.516 | 103.2 | 0.97 |  |
| 1 | 1.032 | 103.3 | 0.75 |  |
