# Supplementary material for: The Conserved Actinobacterial Two-Component System MtrAB Coordinates Chloramphenicol Production with Sporulation in Streptomyces venezuelae NRRL B-65442
Source: Front Microbiol. 2017 Jun 28;8:1145. doi: 10.3389/fmicb.2017.01145 (PMC5487470; doi:10.3389/fmicb.2017.01145)

Supplementary Figure 1. Top: The *Streptomyces coelicolor sco3014-mtrAB-lpqB* operon. Bottom: Distribution of orthologues of *S. coelicolor* SCO3014, MtrA, MtrB and LpqB proteins encoded in more than 100 actinobacterial genomes, as detected by reciprocal BLASTP best hits from http://streptomyces.org.uk/actinoblast/. Each column represents one genome, and the genomes are grouped and coloured to indicate subgroup relationships (e.g. *Corynebacterineae* columns, including *Mycobacterium*, *Nocardia* and *Corynebacterium*, were coloured Indian red). Grey boxes indicate reciprocal hits falling below the minimal criteria adopted for orthology. White boxes indicate the absence of a reciprocal hit. The presence of the rare TTA codon is highlighted by an T in the coloured box (Chandra, G. & Chater, K.F., 2014. Developmental biology of *Streptomyces* from the perspective of 100 actinobacterial genome sequences. *FEMS Microbiol. Lett.*, 38, pp.345–379).

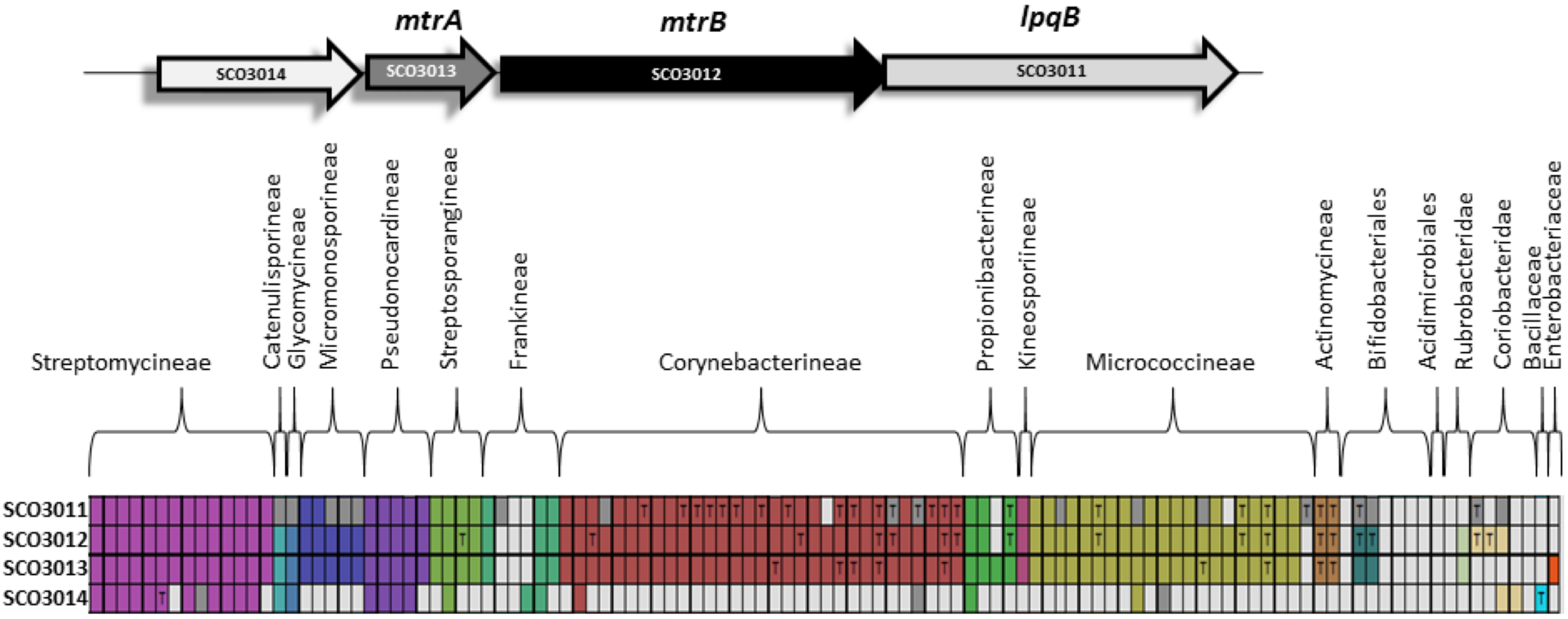

Supplementary Figure 2. All of the strains made and used in this work (Supplementary Table 1) grow like wild-type in liquid and solid agar medium except the Δ*mtrB* mutant which has a mild growth defect on solid agar medium.

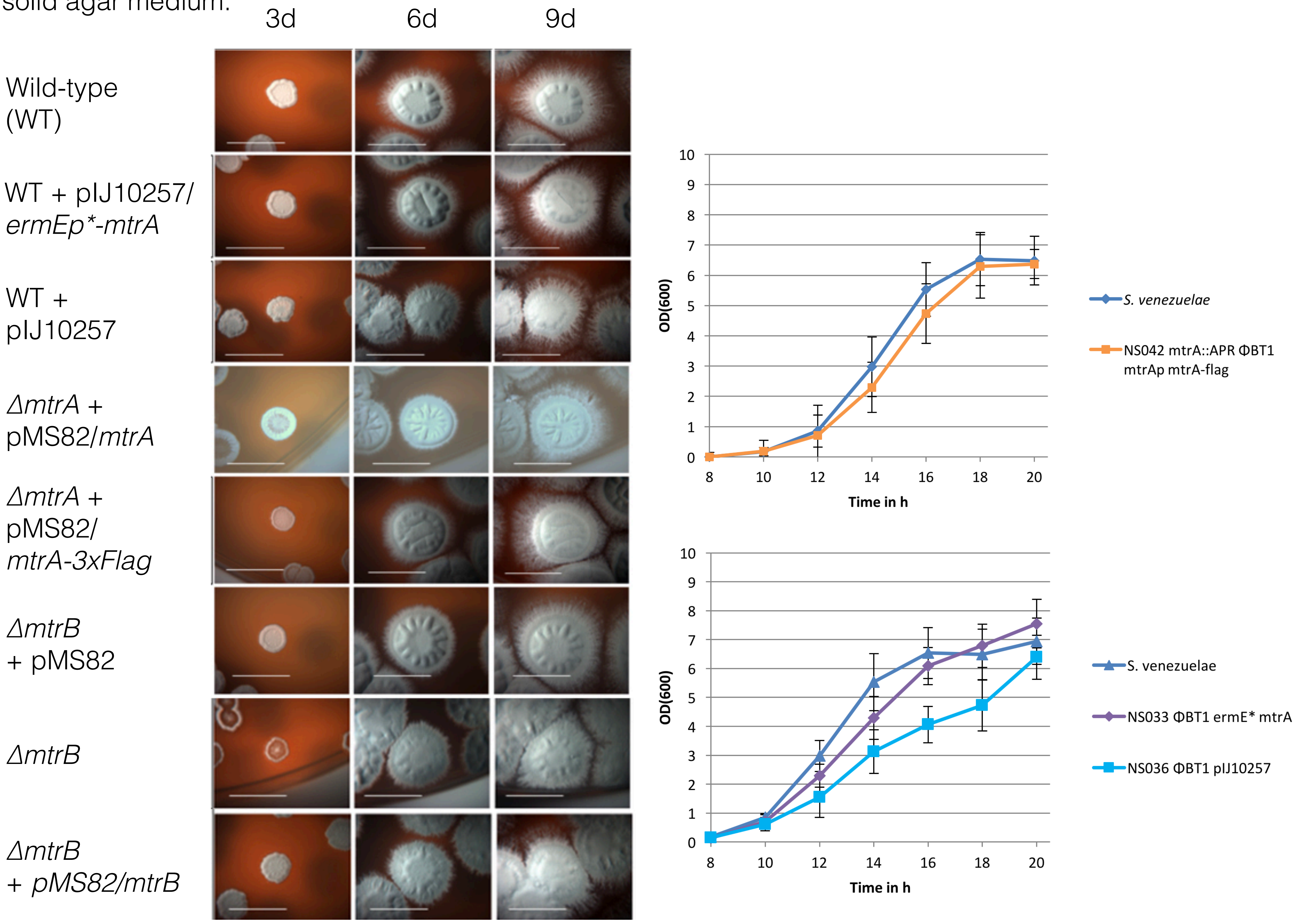

Supplementary Figure 3. Differential RNA sequencing data (dRNA-seq) at 12, 16 and 20 hours growth in liquid MYM medium show two putative transcript start sites for the *mtrAB-lpqB* operon at +1 and -79bp relative to the *mtrA* translational start codon.

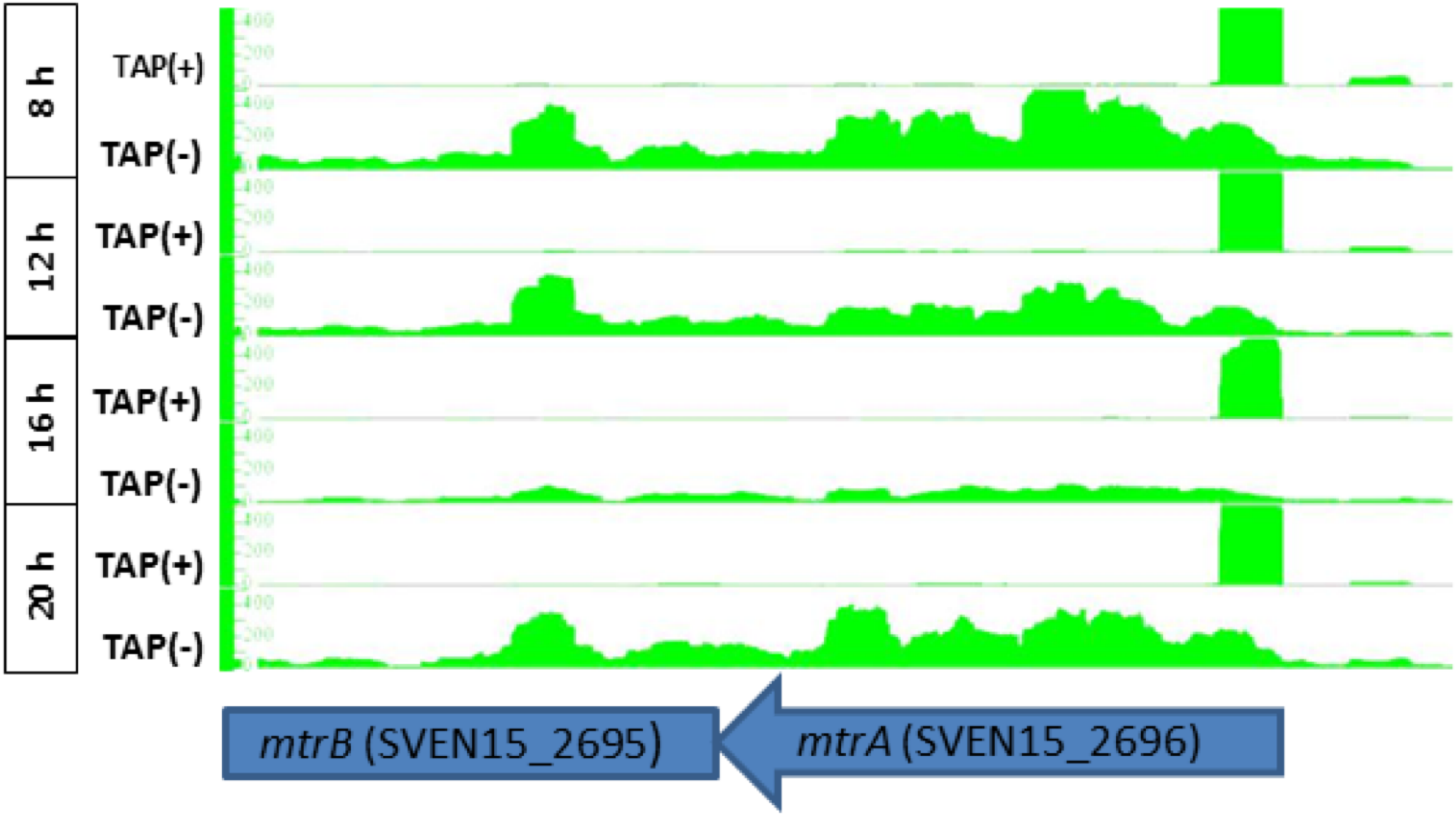

Supplement: Supplementary file 2 [file Image_1.pdf]
