# Supplementary figures and images for: The Conserved Actinobacterial Two-Component System MtrAB Coordinates Chloramphenicol Production with Sporulation in Streptomyces venezuelae NRRL B-65442
Source: Front Microbiol. 2017 Jun 28;8:1145. doi: 10.3389/fmicb.2017.01145 (PMC5487470; doi:10.3389/fmicb.2017.01145)

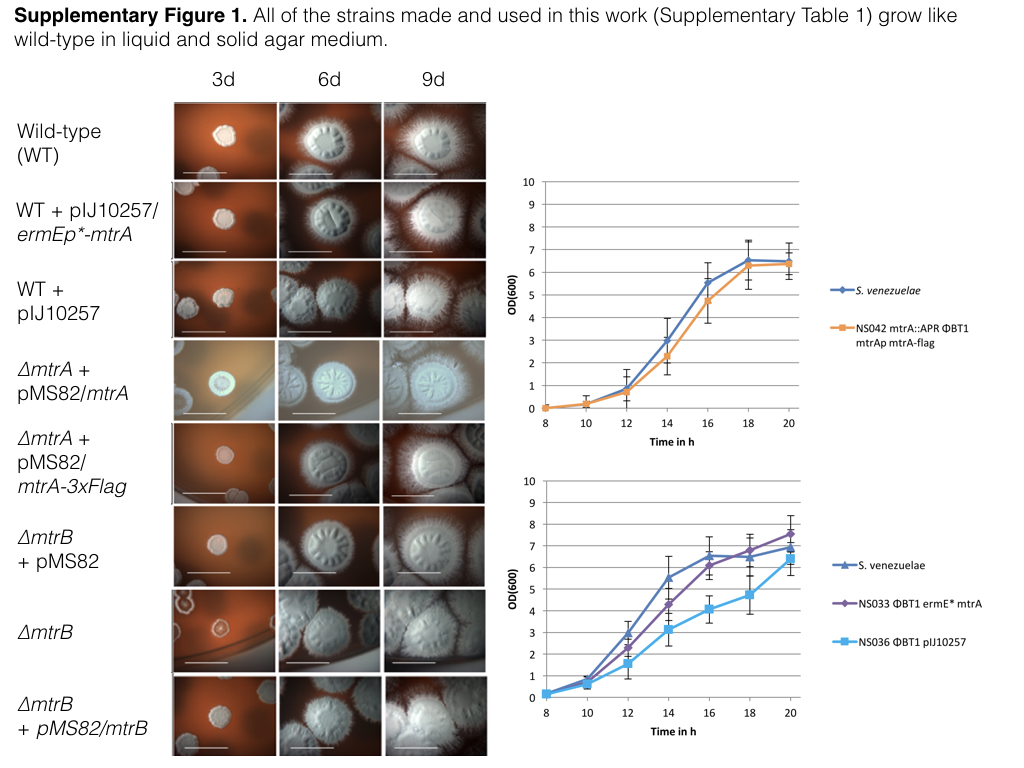

Supplement: Supplementary file 3 [file Image_1.TIFF]

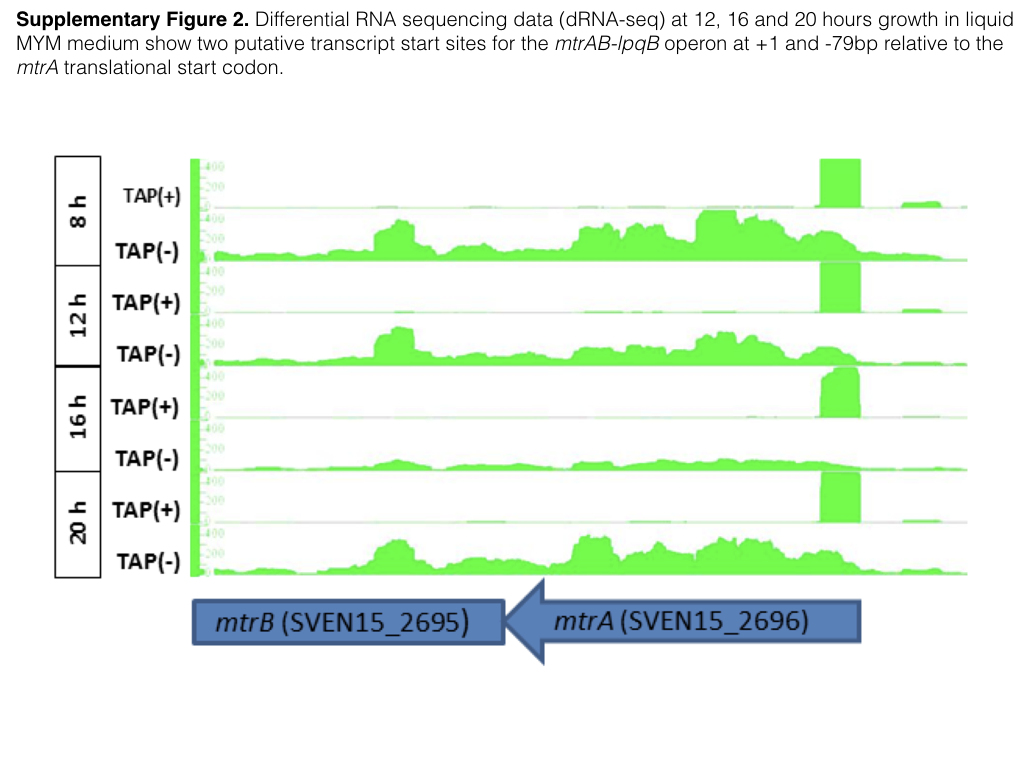

Supplement: Supplementary file 4 [file Image_2.TIFF]

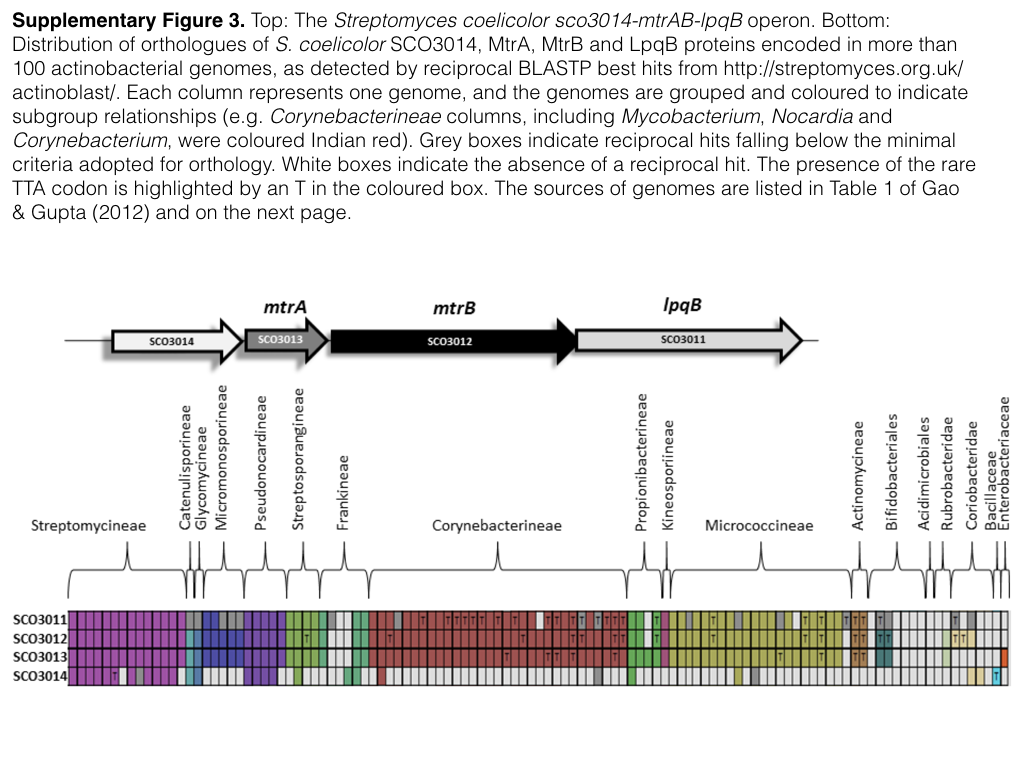

Supplement: Supplementary file 5 [file Image_3.TIFF]
